# Orthopedic Surgery

**Published:** 1906-09

**Authors:** Prescott Le Breton

**Affiliations:** Buffalo


### PROGRESS IN MEDICAL SCIENCE.

### Orthopedic Surgery.

Reported by PRESCOTT LE BRETON, M. D., Buffalo.

BIER'S passive congestion treatment is steadily gaining in popularity, and is now being employed in this country. A summary of recent literature is as follows: Bier uses three means to produce hyperemia,—hot air, constriction and suction. Hot air treatments are reserved for chronic joint cases where there is no infection or where the infection has ceased; for example, old rheumatic joints, arthritis deformans, or gonorrheal joints.

For constriction, a rubber bandage or sometimes a rubber tubing is wound about the extremity on the proximal side of the part to be treated. There are two entirely distinct methods. The first is to apply the constriction for one or two hours a day for tubercular joints. The second to apply the bandage for twenty to twenty-two hours a day for all other kinds of conditions. The rules governing the first method are:

Apply flannel from below up to the joint; above the joint apply a lawer of gauze, and over this the rubber or elastic cotton bandage.

The constriction should produce a venous stasis only; the limb must be warmer than the opposite one, and red. Not cold and blue. No sensation of pins and needles allowed.

Change the area for the constriction if possible; apply the bandage as far as possible from the diseased joint.

If a cold abscess appears, open by a small incision, do not scrape or pack, but change the treatment to the suction cup.

Use no fixation for the joint; begin passive motion almost at once; leg cases require stilting (apparatus to prevent the weight of the body from falling on the affected limb).

The contraindications are amyloid degeneration of the viscera; consumption; the presence of an abscess.

For suppurative cases the suction treatment replaces the constriction. If the abscess has not broken, it is opened and variously sized cups are applied in the following way: a little lanolin is rubbed on the skin about the sinus to make the cup hold well.

The cup is used for five minutes, then taken off for three minutes. This alternation is kept up for forty-five minutes, once daily. The discharge becomes serous and the granulations become red and firm instead of pale and flabby. The treatments are then given every second to fifth day until the sinuses are healed. The hip and knee, on account of the depth of the focus, seem to be the only unfavorable sites for this treatment. Other joints yield rapidly and the results claimed are surprising as regards rapidity of healing, return of motion and of power. The cups are similar to dry cups, with an opening over which is a rubber bulb, which produces the suction; all shapes and sizes are used, to better fit the area involved.

The method of prolonged constriction, to induce a nearly constant passive congestion, is employed for all conditions other than the tubercular, and is combined with the suction treatment by cup, if there is a discharging wound. The constriction is continued for twenty or twenty-two hours out of the twenty-four. Great edema and discoloration are produced, which subside rapidly during the short period of rest, when the part is elevated. The constriction point must be changed often; an inflated rubber tube may be made to replace the Esmarch bandage; about the neck a cotton elastic bandage may be used, and the face becomes bloated and bluish. It is easy to do too much or too little with this prolonged constriction, hence the advice to begin with the furuncles and later undertake cases of greater gravity. For the suppurative cases, incise early making a small opening; use no drainage or packing; cover with a light dressing; apply the suction treatment. Constriction about the neck is advised for cases of diphtheria, sore throat, mastoiditis, erysipelas, jaw abscess, glandular abscess, and the like. In the extremities, constriction also is advised for fractures, suppurative processes, (especially of a superficial nature), teno-vaginitis, gonorrhoeal arthritis.

Reasons for the curative action of the congestion are:

Bactericidal effect of the serum.
Fibrous tissue is more rapidly formed.
Exudates and blood clots are quickly removed after the congestion is discontinued.
The toxines are diluted by the inundation of serum.

Advantages of the method:

It is cheap, easy to use, devoid of danger except where prolonged constriction is given for acute troubles.
It relieves pain markedly.
It prevents ankylosis.
It dispenses with fixation.
A small scar is left, and painful dressings are dispensed with.

Goldthwait and Osgood, (*Boston Medical and Surgical Journal*, May, 28, 1905,) consider the pelvic articulations, from an anatomical, pathological, and clinical standpoint. They are true joints with a slight amount of normal motion, which is increased during pregnancy and in certain other conditions. The causes giving rise to symptoms, are: excess of motion during pregnancy or the menses; traumatism, from falls, or lifting, or the drag of a pendulous abdomen; general lack of tone; infectious diseases; arthritis deformans. The symptoms are: backache, local tenderness, muscle spasm, atrophy of buttock or thigh, and abnormal motion at the symphysis and sacroiliac joints, except in arthritis deformans, where there is no motion obtainable. The backache is referred to the sacrum, over the hips, or down the thigh along the sciatic. It is worse after exercise or on sudden jarring. It is apt to be worse in the night, because in lying on the back the lumbar curve is lessened and strain is brought on the joint. The tenderness is located over the articulations. The muscle spasm limits the normal spinal motions; prevents free forward bending; hinders the raising of the knees as in going up stairs; and may cause the adoption of various postures to relieve the joints.

Abnormal motion may be detected by the following tests: as the patient stands, place the hands over the symphysis and sacrum, and ask him to raise each knee alternately; placing the thumbs on the sacrum and the hands on the crests of the ilium, have the patient repeat the motion; then have the patient lie on the side, and hyperextend the thighs, one at a time, and feel for motion of the ilia on the sacrum. When arthritis deformans has attacked the joint, of course there is no motion. The proximity of the sciatic nerve causes symptoms of sciatica in addition.

Rest and fixation are the principles in treatment. A plaster of paris jacket, often including one thigh by a spica, is the rule. Sometimes rest in bed is advised at first, with a pillow under the lumbar lordosis. Again pressure against the upper sacrum is necessary, or the jacket has to be applied with the patient on his face, so that the sacrum is in normal position. One should remember that during the giving of an anesthetic these joints may suffer strain as the patient lies on a rigid table. Still again a leather jacket, woven trunks, or a pelvic belt will control the symptoms and backache. In the infectious conditions, or tubercular involvements, the joint may have to be opened and scraped. The writers consider that the cases are common, and often overlooked and that the backaches are ascribed to sciatica and rheumatism too often.

